# Angiography-derived assessment of coronary microcirculatory resistance in patients with chronic total occlusion

**DOI:** 10.1007/s00380-025-02619-2

**Published:** 2025-11-17

**Authors:** Michael Molitor, Guilia Gagno, Konstantina Filippou, Maximilian Olschweski, Katrin Steinbach, Markus Vosseler, Zisis Dimitriadis, Philipp Lurz, Philip Wenzel, Tommaso Gori, Recha Blessing

**Affiliations:** 1 Department of Cardiology, University Medical Center Mainz, Johannes Gutenberg University, Langenbeckstr.1, 55131 Mainz, Germany; 2 German Center for Cardiovascular Research (DZHK), Partner Site Rhine-Main, Mainz, Germany; 3 Center for Thrombosis and Hemostasis (CTH), Johannes Gutenberg University, Mainz, Germany; 4 Cardiovascular Department, Department of Medical Surgical and Health Sciences, Azienda Sanitaria Universitaria Di Trieste, University of Trieste, Trieste, Italy

**Keywords:** Chronic total occlusion (CTO), Coronary microvascular dysfunction (CMD), Angiographic Index of microvascular resistance (angio-IMR), Percutaneous coronary intervention (PCI), Coronary artery disease (CAD)

## Abstract

Coronary microvascular dysfunction (CMD) represent a crucial and often underdiagnosed cause of myocardial ischemia and dysfunction. It is closely linked to the prognosis of patients with coronary artery disease. Increased microvascular resistance (whether due to CMD or vascular rarefaction) is more frequent in the setting of coronary chronic total occlusions (CTO). Whether recanalization contributes to the recovery of microvascular function and whether measures of microvascular resistance can potentially be used as prognostic parameter to predict long-term success of CTO recanalization remains unknown. The aim of this study was to investigate CMD in patients with CTO and the effect of successful CTO recanalization. As well, we investigate whether CMD can be identified as a risk factor for restenosis after CTO recanalization. 119 patients underwent successful CTO recanalization at the University Medical Center in Mainz. After a follow-up period of 6 months, invasive control was carried out, in which 79 patients continued to have sufficient revascularization and 40 presented with restenosis. Angiography-based microvascular resistance (Angio-IMR) measurements were performed directly after successful CTO recanalization and at 6 months follow-up offline using a software package (QAngio XA 3D; Medis Medical Imaging Systems). 64% of the patients were male with an average age of 62 ±  9 years. The mean follow-up period was 191 ± 80 days. Median J-CTO Score was 1.8 ± 0.7. The CTO was localized at the RCA in 60%, at the LAD in 20% and at the LCX in 24% of the patients. All included patients had a good result after CTO recanalization confirmed by Quantitative flow ratio (QFR) of 0.94 ± 0.04 directly after PCI. Angio-IMR values immediately after CTO recanalization were pathological (> 25) in 78% of the patients and showed a significant decrease at 6 months follow-up (31.7 ± 7.1 vs. 28.6 ± 5.3¸ *p* = 0.0024). Post-procedural angio-IMR values did not predict restenosis at 6-month follow-up (31.7 ± 8 vs. 29.8 ± 7.5, *p* = 0.173). CMD can be detected in a majority of patients after successful CTO PCI. At 6 months follow-up we found significant improved angio-IMR values; CMD was not a predictor of restenosis.

## Introduction

Despite advancements in risk stratification, diagnostics, and preventive measures, coronary artery disease (CAD) remains one of the most prevalent diseases and a leading cause of mortality worldwide [1]. Chronic total occlusion (CTO) of a coronary artery, defined as a complete stenosis with Thrombolysis In Myocardial Infarction (TIMI) grade 0 flow persisting for more than three months, is found in approximately 15–25% of patients undergoing coronary angiography [2].

Recanalization of CTOs is considered a complex procedure and poses significant challenges for interventional cardiologists [3, 4], but studies indicate that successful recanalization is associated with improved health status and quality of life due to symptom relief [5–9]. Nonetheless, the prognostic benefits and survival impact remain subjects of ongoing debate [10–14]. Additionally, complication and restenosis rates for CTO recanalization exceed those observed in standard interventions, highlighting the critical importance of optimal patient selection to determine who may derive the greatest benefit from the procedure or alternative therapeutic approaches.

Coronary microvascular dysfunction (CMD) plays a pivotal role in the pathogenesis of cardiovascular diseases. CMD arises from multifactorial mechanisms, including impaired vasomotor function, microvascular obstruction, microvascular injury and capillary rarefaction. It can be evaluated through invasive and non-invasive modalities, with the invasive index of microcirculatory resistance (IMR) being the most widely used metric [15, 16]. The gold standard for CMD assessment involves invasive techniques utilizing pressure-wire-based and thermodilution-derived indices. Recently, technological advancements have facilitated the development of non-invasive methods to calculate IMR from angiographic images [15, 17, 18]. Studies have demonstrated that angiography-derived IMR (angio-IMR) is a feasible and accurate approach for estimating coronary microcirculatory resistance [18–22].

Currently, limited data exist regarding the presence of CMD in patients with CTO. Emerging evidence suggests that CMD is prevalent among patients with CTO [23–26]. The prognostic value of microvascular dysfunction has already been established in various cardiovascular conditions, including CAD, Takotsubo cardiomyopathy, and heart failure [1, 20, 27, 28].

This study aims to evaluate the role of CMD in patients with CTO and investigate whether CMD serves as a risk factor for restenosis following successful CTO recanalization.

## Methods

### Study design

A retrospective, monocentric analyzation of the CTO database of the University Medical Center in Mainz was performed for patients that successfully underwent recanalization of a CTO lesion and surveillance coronary angiography at 6-month follow-up. The database included demographic, clinical, angiographic, and periprocedural information, along with in-hospital and in part long-term outcomes. This research study was conducted retrospectively from data obtained for clinical purposes The Ethics Committee of the University Medical Center Mainz has confirmed that no ethical approval is required.

In the period from 2018 to 2023, we identified 119 patients to include in this study. Exclusion criteria were patients with prior cardiac bypass surgery or insufficient image quality e.g. low contrast medium flow.

## Angiographic IMR

CTO procedures were performed by experienced operators. The CTO hybrid algorithm was used in all cases [29]. Intracoronary nitrates were administered in all patients. Angio-IMR was assessed in the affected CTO vessel after successful recanalization and at an invasive 6 months follow-up. CMD was defined as an IMR ≥ 25, in accordance with established validation studies and expert consensus in non-CTO populations. This threshold corresponds to the upper limit of the normal IMR distribution derived from reference cohorts with angiographically and functionally normal coronary arteries and has been consistently associated with adverse outcomes across multiple studies [30–33]. CTO-revascularization, dedicated validation of IMR thresholds is limited; therefore, the same ≥ 25 cutoff was applied by extrapolation, consistent with prior CTO investigations [34–36].

Restenosis at 6 months was defined angiographically by experienced interventional cardiologists as a ≥ 50% diameter stenosis. The angio-IMR analysis were performed by two independent, experienced and certified investigators with a validated software (QAngio XA 3D; Medis Medical Imaging Systems). The assessment of quantitative flow ratio (QFR) was performed following current practice described in the Favor II Europe-Japan study [37].

We performed Medis QFR® analysis offline choosing two angiographic projections at least 25° apart with sufficient quality (good contrast agent flow, avoiding foreshortening and overlap). The end-diastolic frame was selected automatically, was checked and corrected manually if necessary. Anatomical landmarks (e.g. bifurcations) clearly visible in both projections were selected as reference points and proximal und distal points were marked. The contour of the vessel was automatically recognized based on the markers set and corrected manually if necessary. The frame where the contrast agent flows into the area of the proximally placed marker and the frame where the contrast agent reaches the area of the distally placed marker were assessed manually. As a final step a 3D vessel reconstruction is created on the basis of the data and QFR/angio-IMR are calculated by the software. The formulas implemented in our Software are described by Zhou et al. [22].

## Statistical analysis

Statistical analyses were performed using SPSS (Version 29, IBM SPSS Statistics) and GraphPad Prism software, version 10 (Graph Pad Software Inc., La Jolla, CA, USA). Normal distribution was assessed with the Kolmogorov–Smirnov-Test. Categorial variables are presented as count and percentage and analysed by Chi Square test or Fisher exact test. Continuous variables with normal distribution are reported as mean and standard deviation and comparison between groups was performed using Student`s* t* test. Variables with non-normal distribution are presented as median with interquartile range and analysed by Mann–Whitney U-Test. In order to investigate the effect of microvascular dysfunction on the risk of restenosis, the cohort was divided into 2 groups (restenosis vs. no-restenosis). Angio-IMR mean and standard deviation between the two groups were performed by Student`s* t* test.

**Fig. 1:**
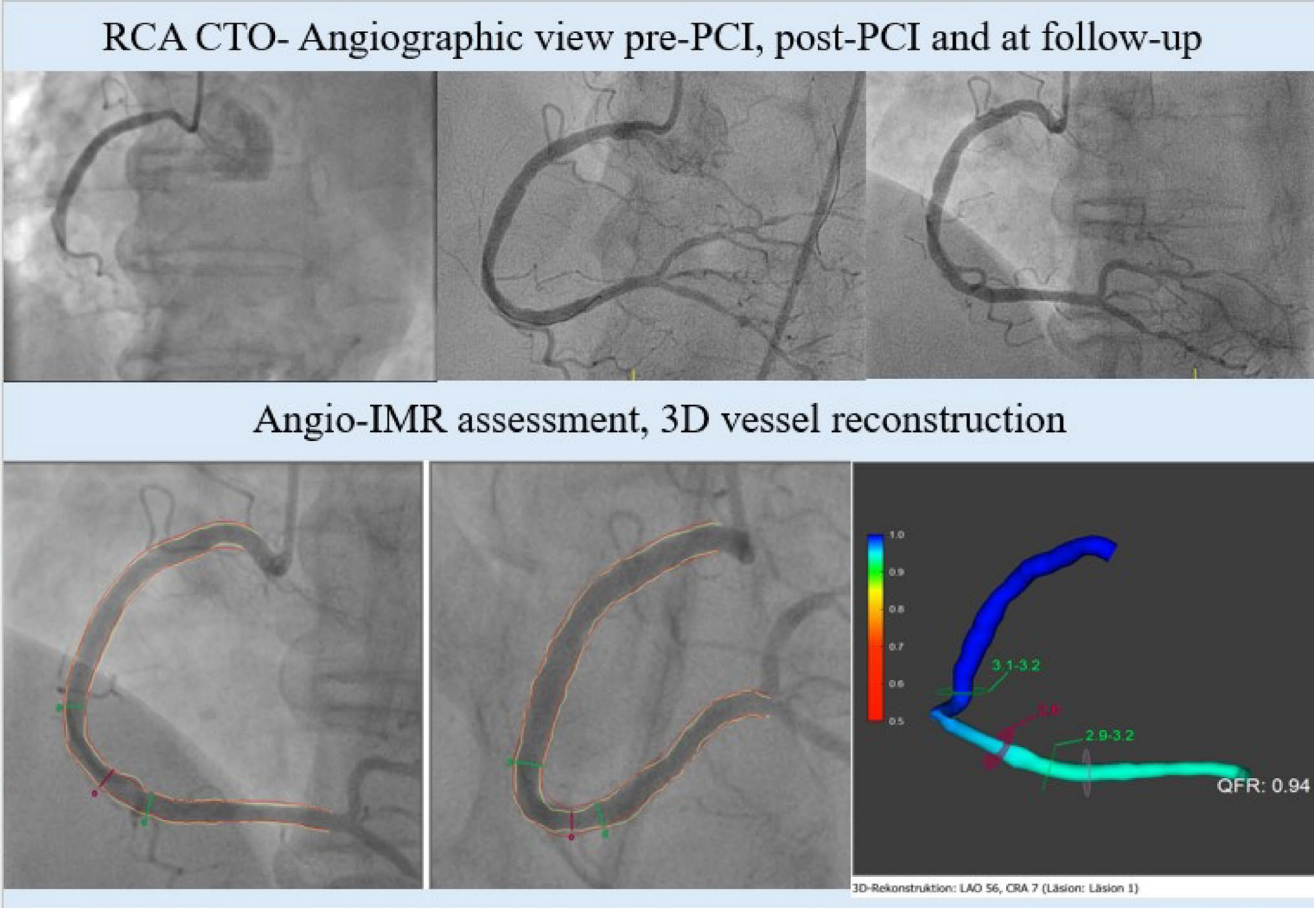
Exemplary visualization of the study workflow. *Top row:* Successful recanalization of a CTO of the right coronary artery (RCA); *Bottom row*: Diagnostic Algorithm of the Angio-IMR analysis

A* p* value < 0.05 was considered statistically significant (Fig. 1).

## Results

### Demographic and clinical baseline characteristics

Demographic parameters at baseline are shown in Table 1. In our cohort 62% were male with a median age of 62 ± 5 years. The mean follow-up period was 191 ± 80 days. Median J-CTO Score was 1.8 ± 0.7 and most of the patients (70.6%) had Rentrop grade 2 of coronary collateral circulation.

**Table 1:** Baseline characteristics of the study population

	All patients (n = 119)
*Demographics characteristics*	
Age, yrs	62.45 $\pm$ 9.21
Male	76 (63.9)
BMI, kg/m^2^	27.68 (16.14- 35.64)
Diabetes mellitus	19 (16.0)
Hypertension	106 (89.1)
Hyperlipidemia	89 (74.8)
Current smoking	39 (32.8)
Multivessel CAD	93 (78.2)
GFR, ml/min	79.00 (36.00–114.00)
LVEF, %	55.00 (25.00–65.00)
Previous stroke	9 (7.6)
PAD	13 (10.9)
Previous MI	36 (30.3)
Previous PCI	84 (70.60)
*Medication*	
ACE-I	65 (54.6)
AT1R-B	30 (25.2)
ARNI	9 (7.6)
BB	76 (63.9)
MRA	26 (21.8)
CCB	28 (23.5)
Statins	100 (100)
*Procedural characteristics* *CTO vessel*	
RCA	71 (59.7)
LAD	24 (20.2)
LCX	24 (20.2)
J-CTO Score	1.81 ± 0.73
Total stent length, mm	55.9 ± 22.01
QFR	0.94 ± 0.04
IMR baseline	31.09 ± 7.33

Values are represented as n (%), median (interquartile range), or mean ± SD

yrs, years; BMI, body mass index; CAD, coronary artery disease; GFR, glomerular filtration rate; LVEF, left ventricular ejection fraction; PAD, peripheral artery disease; MI, myocardial infarction; PCI, percutaneous coronary intervention; ACE-I, angiotensin-converting enzyme inhibitor; AT1R-B, angiotensin II type-1 receptor blocker; ARNI, angiotensin receptor–neprilysin inhibitor; BB, beta-blocker; MRA, mineralocorticoid receptor antagonist; CCB, calcium channel blocker; CTO, chronic total occlusion; QFR, quantitative flow ratio; IMR, index of microcirculatory resistance

In order to detect possible differences in the patient cohorts with and without detected CMD, the baseline characteristics were compared, which are shown in Table 2. We did not find any significant differences between the two groups with the same prevalence of cardiovascular risk factors and comorbidities. There was a high proportion of patients with arterial hypertension and hypercholesterolemia in both groups. We were also unable to detect any differences in the medication of the patients with ACE inhibitors/AT1 receptor blockers/ARNIs, beta blockers, calcium channel antagonists, mineralocorticoid receptor antagonists or statins that potentially impact microvascular function.

**Table 2:** Characteristics of patients with and without MVD

	MVD (n = 93)	No MVD (26)	p value
*Demographics characteristics*			
Age, yrs	62.12 ± 9.21	63.65 ± 9.33	0.46
Male	77 (82.8)	22 (84.6)	0.82
BMI, kg/m^2^	27.68 (16.14–35.50)	28.01 (21.60–34.64)	0.45
Diabetes mellitus	16 (17.2)	3 (11.5)	0.48
Hypertension	81 (87.1)	25 (96.2)	0.19
Hyperlipidemia	71 (76.3)	18 (69.2)	0.46
Current smoking	30 (32.3)	9 (34.6)	0.82
Multivessel CAD	74 (79.6)	19 (73.1)	0.47
GFR, ml/min	80.02 ± 15.77	77.46 ± 14.64	0.45
LVEF, %	55.00 (25.00–65.00)	55.00 (25.00–55.00)	0.97
Previous stroke	5 (5.4)	4 (15.4)	0.08
PAD	10 (10.8)	3 (11.5)	0.91
Previous MI	29 (31.2)	7 (26.9)	0.67
Previous PCI	66 (71)	18 (69.2)	0.86
*Medication*			
ACE-I	53 (57.0)	12 (46.2)	0.32
AT1R-B	23 (24.7)	7 (26.9)	0.82
ARNI	6 (6.5)	3 (11.5)	0.39
BB	57 (61.3)	19 (73.1)	0.27
MRA	18 (19.4)	8 (30.8)	0.21
CCB	22 (23.7)	6 (23.1)	0.95
Statins	93 (100)	26 (100)	1.00
*Procedural characteristics* *CTO vessel*			
RCA	54 (58.1)	17 (65.4)	0.50
LAD	20 (21.5)	4 (15.4)	0.49
LCX	19 (20.4)	5 (19.2)	0.89
J-CTO Score	2 (1–3)	2 (0–3)	0.31
Total stent length, mm	56.37 ± 22.26	54.16 ± 21.40	0.65
QFR	0.98 (0.73–1.00)	0.99 (0.91–1.00)	0.29
IMR baseline	33.66 ± 6.10	21.89 ± 1.99	< 0.001

Values are represented as n (%), median (interquartile range), or mean ± SD. MVD, microvascular dysfunction; yrs, years; BMI, body mass index; CAD, coronary artery disease; GFR, glomerular filtration rate; LVEF, left ventricular ejection fraction; PAD, peripheral artery disease; MI, myocardial infarction; PCI, percutaneous coronary intervention; ACE-I, angiotensin-converting enzyme inhibitor; AT1R-B, angiotensin II type-1 receptor blocker; ARNI, angiotensin receptor–neprilysin inhibitor; BB, beta-blocker; MRA, mineralocorticoid receptor antagonist; CCB, calcium channel blocker; CTO, chronic total occlusion; QFR, quantitative flow ratio; IMR, index of microcirculatory resistance

### Angiography-derived index of microcirculatory resistance

**Fig. 2:**
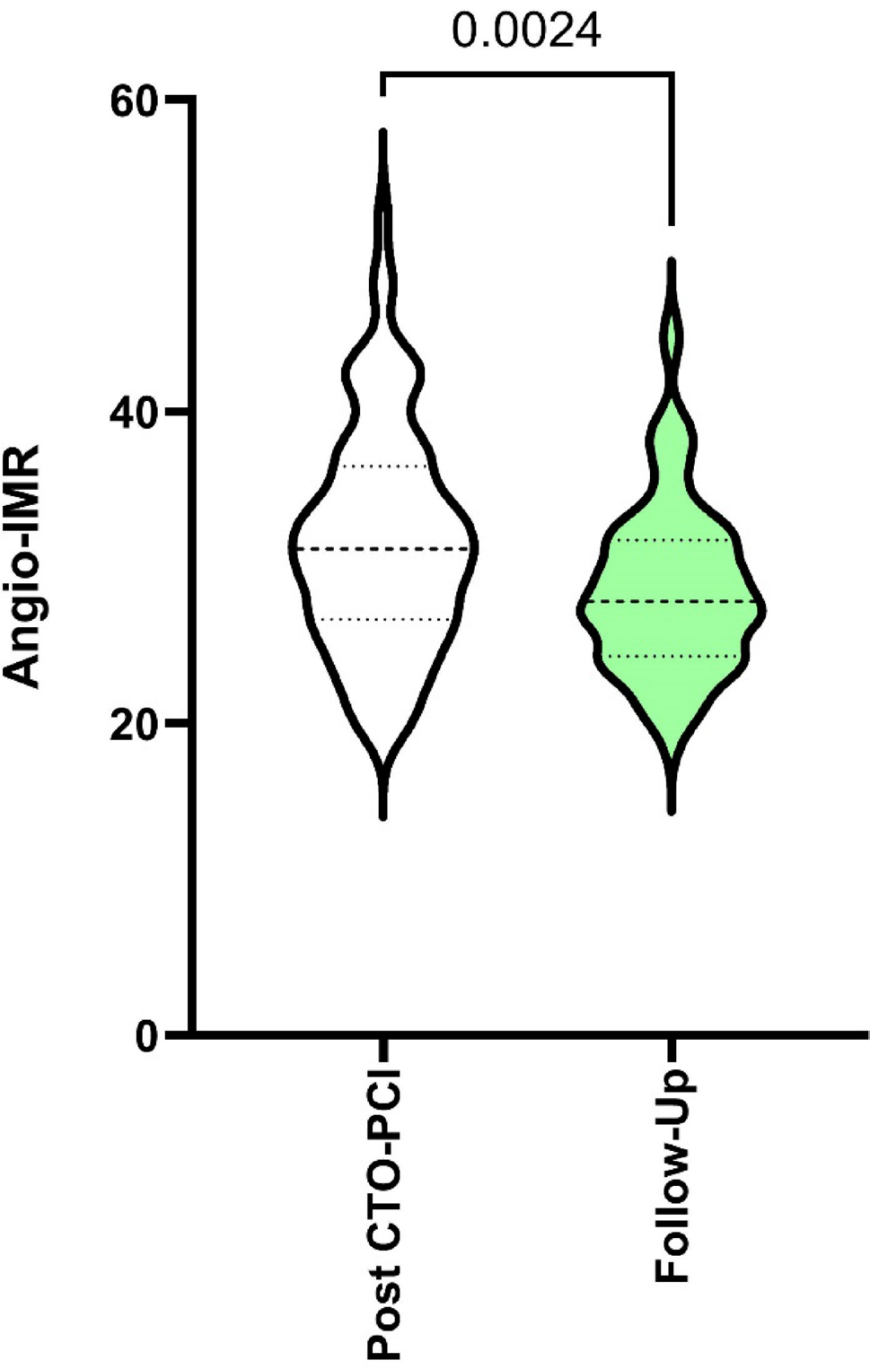
Angio-IMR values immediately after successful CTO-PCI as well as after a 6-month follow-up (n = 79)

An elevated angio-IMR value (> 25) was found in 93 patients (78%) immediately after successful revascularization of a CTO vessel in our total cohort of 119 patients. Elevated angio-IMR was present in 57 of 79 (72%) patients with a long-term revascularized CTO vessel at the 6-months follow-up with a significant decrease (from 31.7 ± 8 vs. 29.8 ± 7.5, *p* < 0.0024). The results are shown in Fig. 2.

**Fig. 3:**
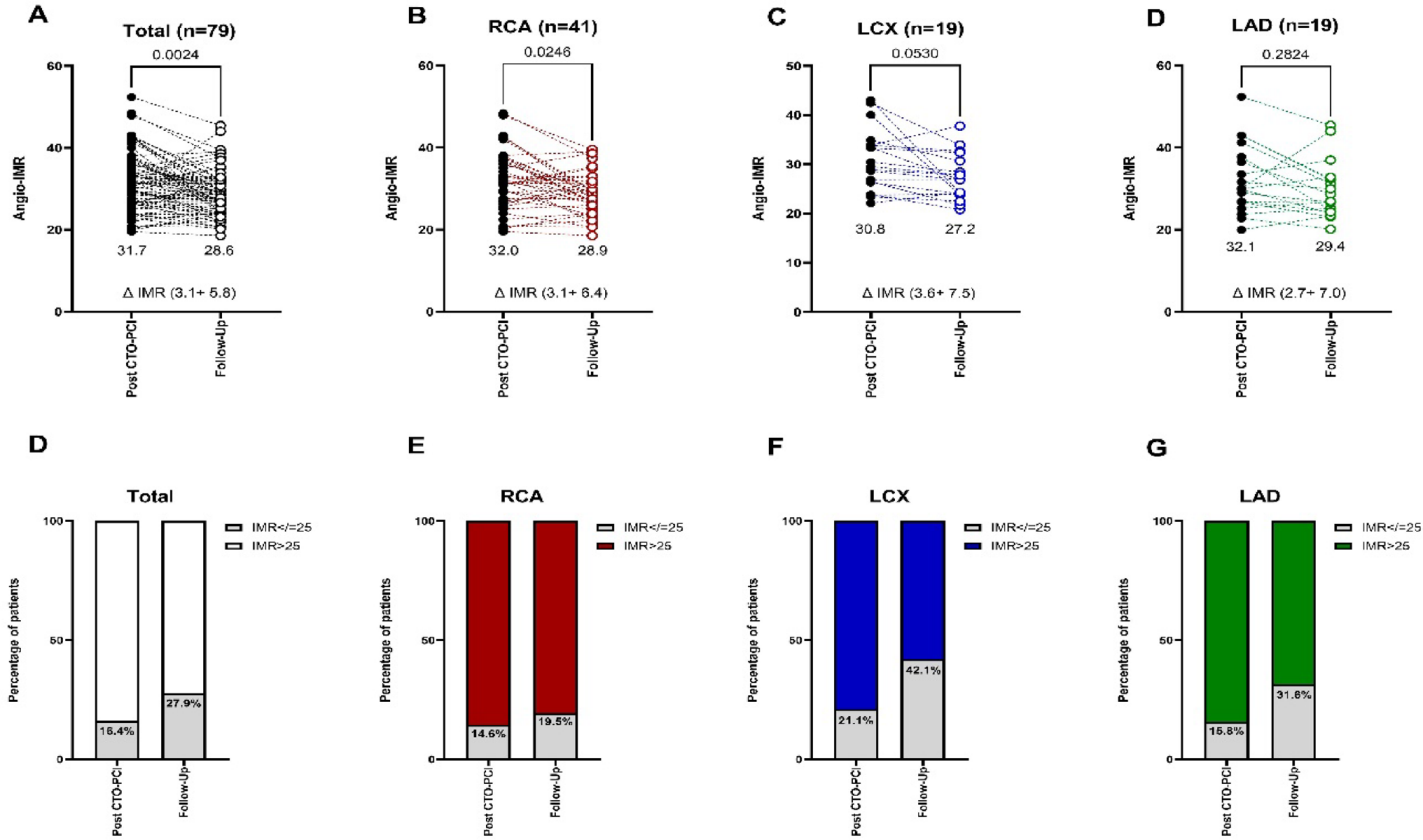
Angiography-derived index of microcirculatory resistance (angio-IMR) immediately after successful CTO-PCI and at 6-month follow-up in persistent revascularized CTO vessels. **A** Total patient cohort (n = 79); **B** RCA CTOs (n = 41); **C** LCX CTOs (n = 19); **D** LAD CTOs (n = 19). Mean angio-IMR values as well as Δ IMR + SD are shown below each panel, and *p*-values indicate paired comparisons between post-CTO-PCI and follow-up. Panels **E**–**G** depict the proportion of vessels with angio-IMR ≥ 25 and ≤ 25 at each time point, stratified by coronary territory

In-depth analysis of the three coronary territories demonstrated a numerical reduction in angio-IMR across all vessels 6 month after persistent successful CTO-PCI without restenosis. The decrease reached statistical significance in RCA CTOs only (Δ IMR: 3.1 ± 6.4; *p* = 0.0246), while reductions in LAD (Δ IMR: 2.7 ± 7.0; *p* = 0.283) and LCX (Δ IMR: 3.6 ± 7.5; *p* = 0.053) were of similar magnitude but did not reach statistical significance, likely reflecting smaller sample sizes in these subgroups. When analysed categorically using the established threshold of angio-IMR ≥ 25, the proportion of vessels with abnormal microvascular resistance decreased from 83.6 to 72.1% in the overall cohort, with consistent numerical improvement across all coronary territories (RCA: 85.4 to 80.5%; LCX: 78.9 to 57.9%; LAD: 84.2 to 68.4%). Notably, a successful revascularization was most frequently associated with an improvement from angio-IMR > 25 to ≤ 25 in the left coronary arteries (LAD and LCX). This observation may reflect the larger myocardial perfusion territories and greater potential for microvascular recovery in the left coronary circulation following relief of long-standing ischemia. Overall, 32.9% of patients transitioned from angio-IMR > 25 to ≤ 25 at follow-up, indicating partial recovery of microvascular function after CTO-PCI (Fig. 3).

### Relationship between microvascular resistance and restenosis

**Fig. 4:**
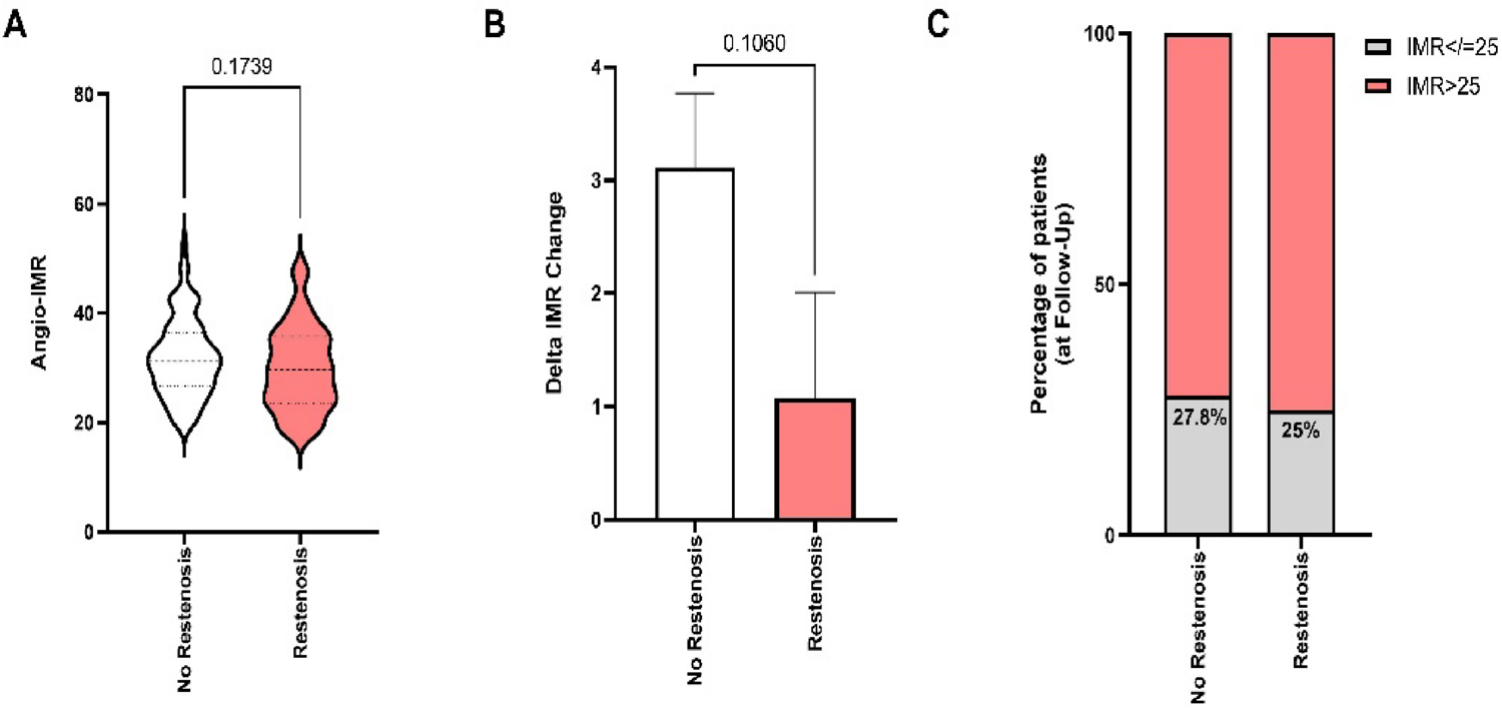
Relationship between angiography-derived index of microcirculatory resistance (angio-IMR) and restenosis at 6-month follow-up. **A** Angio-IMR values immediately after successful CTO-PCI stratified by restenosis status. **B** Change in angio-IMR (ΔIMR = follow-up—post-PCI) comparing no-restenosis and restenosis groups. **C** Proportion of patients with angio-IMR ≥ 25 and ≤ 25 at follow-up, stratified by restenosis status. Bars and violin plots display mean ± SD and distribution; *p*-values correspond to unpaired comparisons between groups

At 6-month follow-up, 79 of 119 patients (66.4%) showed no angiographic restenosis, whereas 40 patients (33.6%) presented with binary restenosis. Immediately after successful CTO-PCI, angio-IMR values were comparable between both groups (no restenosis: 31.7 ± 7.1 vs. restenosis: 29.8 ± 7.5; *p* = 0.17; Fig. 4A). To further explore whether restenosis influenced microvascular function over time, we compared the change in angio-IMR (Δ IMR) between the two groups. The reduction in IMR from baseline to follow-up was numerically greater in the no-restenosis group (Δ IMR = 3.0 ± 6.6) than in patients with restenosis (Δ IMR = 1.4 ± 5.8), but this difference did not reach statistical significance (*p* = 0.11; Fig. 4B). Similarly, at follow-up, the proportion of patients with persistent CMD (angio-IMR ≥ 25) was comparable between groups (no restenosis: 27.8% vs. restenosis: 25.0%; Fig. 4C).

Collectively, these data indicate that microvascular resistance and its longitudinal improvement are largely independent of angiographic restenosis, suggesting that CMD and restenosis represent distinct pathophysiological processes after successful CTO revascularization.

### Clinical impact of successful CTO recanalization

**Fig. 5:**
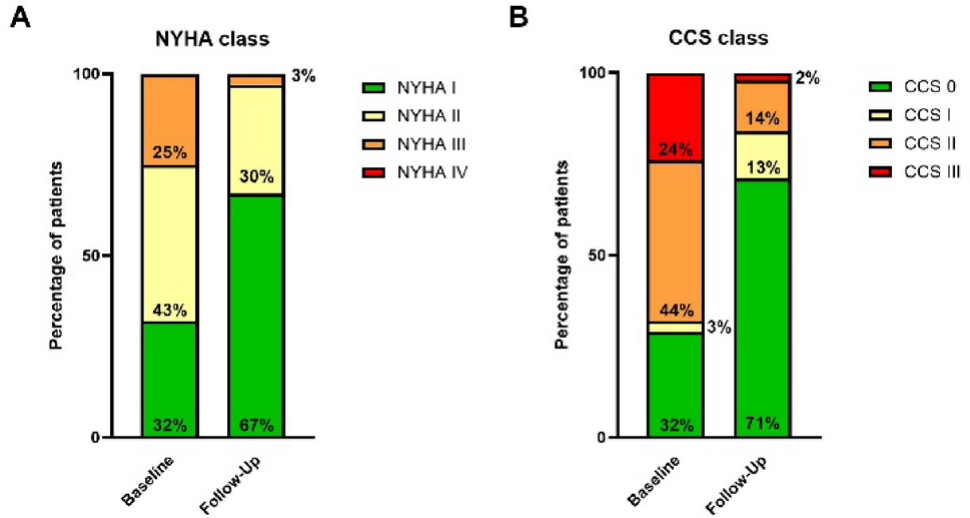
Changes in symptom burden and functional capacity following successful CTO-PCI. **A** Distribution of patients according to NYHA functional class at baseline and 6-month follow-up. **B** Distribution of CCS angina class at the same time points. n = 119

As illustrated in Fig. 5, successful CTO-PCI was associated with a marked improvement in clinical status. Overall, 55% of patients demonstrated an improvement in Canadian Cardiovascular Society (CCS) angina class, and 46% showed an improvement in New York Heart Association (NYHA) functional class at 6-month follow-up. Complete freedom from angina (CCS 0) was achieved in 71% of patients, and 67% reported no limitation of physical activity (NYHA I), reflecting substantial symptomatic and functional recovery after recanalization. When stratified by the presence of CMD, improvement in both CCS and NYHA class did not differ between patients with or without microvascular dysfunction (*p* = 0.459 and *p* = 0.418, respectively), indicating that symptomatic benefit after successful CTO-PCI occurs largely independent of microvascular resistance status.

## Discussion

We investigated the clinical significance of CMD in patients with CTO using non-invasive angio-derived IMR (angio-IMR) measurements. Our main findings were as follows: (I) patients with CTO lesions exhibit elevated IMR values indicative of CMD immediately after successful revascularization; (II) persistent reperfusion of the CTO vessel results in a significant reduction of IMR values at the 6-month follow-up, accompanied by symptomatic improvement in NYHA and CCS classifications; and (III) neither baseline IMR values nor their improvement were predictive of restenosis after CTO-PCI. In addition, we observed that successful CTO-PCI led to a highly significant improvement in both CCS and NYHA class, and that this clinical benefit occurred independently of the presence of CMD (no difference in CCS or NYHA improvement between IMR > 25 and ≤ 25). These results suggest that while microvascular recovery accompanies revascularization, symptom relief is primarily driven by epicardial flow restoration rather than baseline microvascular status.

CMD has been extensively studied in various clinical conditions of CAD, particularly in acute myocardial infarction (AMI). Elevated IMR values (> 40) have been identified as robust predictors of adverse outcomes, including increased major adverse cardiovascular events (MACE), following ST-elevation myocardial infarction (STEMI) [15, 19, 38–40]. Post-PCI risk stratification using IMR can help identify patients at high risk of cardiac death upon discharge [41]. Furthermore, CMD is prognostically relevant in other conditions including Tako-Tsubo cardiomyopathy and dilated cardiomyopathy [27, 40, 42]. However, research on CMD in the context of stable CAD, particularly in patients with CTO, remains limited.

Microvascular dysfunction can be assessed through both non-invasive (e.g., echocardiography, cardiac magnetic resonance, PET, CT) and invasive methods (e.g., intracoronary Doppler or thermodilution techniques during angiography). Thermodilution is widely used, allowing simultaneous assessment of epicardial stenosis and microvascular dysfunction without requiring additional modalities. However, invasive techniques carry risks, are procedurally complex, and require significant time [43–46].

Angiographic IMR offers a promising alternative by simplifying CMD assessment and allowing its assessment off-line. Validation studies have demonstrated strong correlation between angio-IMR and wire-based IMR (Fan et al.: r = 0.83, *p* < 0.001; diagnostic accuracy: 87.2%; 95% CI 83.0–91.3%) [47]. Similarly, De Maria et al. confirmed the method’s reliability in STEMI patients pre- and post-PCI. Mejía-Rentería et al. [17] further validated the accuracy and feasibility of angio-IMR in patients with suspected myocardial ischemia and non-obstructive coronary arteries [48]. Thus, angio-IMR represents a rapid, cost-effective, and convenient approach to CMD assessment without pressure-wire use.

Our study builds upon limited existing evidence regarding CMD in CTO lesions. Werner et al. highlighted the frequent association between CTO and CMD [7, 8, 26]. Similarly, Keulards et al. demonstrated significant CMD in the myocardium supplied by CTO immediately post-recanalization, with notable improvement at two-month follow-up [49]. These findings align with our observation that CMD was present in 76% of patients at baseline. CTO and CMD may share several pathophysiological links, including shared cardiovascular risk factors, chronic ischemia, and histological myocardial changes. Persistent ischemia, reported in 85% of patients with CTO in the COURAGE trial, induces cellular-level changes, such as immune cell activation, increased inflammation, apoptosis, and vascular remodelling [50–52]. These processes impair vasomotor responsiveness and endothelial barrier integrity—key factors in CMD pathogenesis [51, 53–55].

Comparing our two cohorts, we were unable to find any differences between the patients with regard to the baseline characteristics or medical therapy. These results are most likely due to the fact that CMD and CHD share the same risk factors. We also found no difference in clinical parameters (CCS and NYHA) between the two groups; in our cohort, both groups benefited from successful recanalization as evidenced by an improvement in CCS and NYHA classification.

While CMD has been linked to adverse outcomes in other CAD contexts, its prognostic significance in CTO remains unclear. In our cohort, we were able to demonstrate that the vast majority (78%) of patients with a CTO had elevated IMR values and signs of CMD. A successful and persistent revascularized CTO vessel leads to an improvement of the angio-IMR value in all three coronary vessels. Even though we were only able to prove significance for RCA due to the small group size.

In contrast, CMD did not show a predictive value on the long-term success of a CTO revascularization. Neither the absence of CMD (here with evidence of an angio-IMR value < / = 25) nor the angio-IMR value immediately after successful revascularization were associated with outcomes, while diabetes mellitus and a longer stent distance had a negative impact [56]. Still, our results suggests that CMD may not serve as a reliable predictor for restenosis in CTO patients.

Taken together, our findings indicate that successful CTO-PCI leads to both significant symptomatic recovery and measurable improvement in microvascular function, but that these two phenomena may evolve through distinct physiological mechanisms. Restoration of epicardial flow alleviates ischemic burden and symptoms, whereas gradual microvascular recovery reflects downstream remodeling over time.

## Limitations

This study has several limitations. It was retrospective in design and included a small, monocentric cohort. A significant proportion of patients were excluded due to suboptimal angiographic quality or inadequate views for three-dimensional vessel reconstruction. Furthermore, IMR was measured immediately post-PCI, a setting that may overestimate microvascular resistance due to procedure-related vascular trauma and altered flow dynamics [57]. The definition of pathological IMR as ≥ 25 was originally validated in non-CTO settings. Although this cut-off is widely accepted for identifying coronary microvascular dysfunction and has been adopted in several CTO studies, we acknowledge that its direct validation in the CTO context remains limited [34, 35, 49]. The absence of CFR data and unassessed factors such as residual dissections may also limit interpretation. Our findings should therefore be interpreted with this consideration in mind. In addition, the 6-month follow-up period may not be sufficient to capture the full prognostic implications of CMD or restenosis, particularly regarding long-term clinical outcomes. Although CMD did not predict restenosis in our analysis, other clinically relevant endpoints such as target vessel failure, angina recurrence, or hospitalization were not assessed and may be more sensitive to CMD. Our findings are hypothesis-generating and warrant validation in a prospective study with a larger population.

## Data Availability

All data that support the finding of this study are available from the corresponding author upon request.

## Abbreviation

ACE-I: Angiotensin-converting enzyme inhibitor
AMI: Acute myocardial infarction
ARNI: Angiotensin receptor–neprilysin inhibitor
AT1R-B: Angiotensin II type-1 receptor blocker
BB: Beta-blocker
BMI: Body mass index
CAD: Coronary artery disease
CCB: Calcium channel blocker
CCS: Canadian Cardiovascular Society
CFD: Computational fluid dynamics
CHD: Coronary heart disease
CMD: Coronary microvascular dysfunction
CT: Computed tomography
CTO: Chronic total occlusion
CFR: Coronary flow reserve
DM: Diabetes mellitus
ECG: Electrocardiogram
FFR: Fractional flow reserve
GFR: Glomerular filtration rate
IMR: Index of microcirculatory resistance
IVUS: Intravascular ultrasound
J-CTO Score: Japanese chronic total occlusion score
LAD: Left anterior descending artery
LCX: Left circumflex artery
LVEF: Left ventricular ejection fraction
MACE: Major adverse cardiovascular events
MI: Myocardial infarction
MRA: Mineralocorticoid receptor antagonist
MRI: Magnetic resonance imaging
NYHA: New York Heart Association
OCT: Optical coherence tomography
PAD: Peripheral artery disease
PCI: Percutaneous coronary intervention
PET: Positron emission tomography
QFR: Quantitative flow ratio
RCA: Right coronary artery
STEMI: ST-elevation myocardial infarction
TIMI: Thrombolysis in myocardial infarction

## References

[1] Duggan JP, Peters AS, Trachiotis GD, Antevil JL (2022) Epidemiology of coronary artery disease. Surg Clin North Am 102(3):499–516 35671770 10.1016/j.suc.2022.01.007

[2] Galassi AR, Werner GS, Boukhris M, Azzalini L, Mashayekhi K, Carlino M, Avran A, Konstantinidis NV, Grancini L, Bryniarski L, Garbo R, Bozinovic N, Gershlick AH, Rathore S, Di Mario C, Louvard Y, Reifart N, Sianos G (2019) Percutaneous recanalisation of chronic total occlusions: 2019 consensus document from the EuroCTO Club. EuroIntervention 15(2):198–208 30636678 10.4244/EIJ-D-18-00826

[3] Blessing R, Buono A, Ahoopai M, Geyer M, Knorr M, Brandt M, Steven S, Drosos I, Muenzel T, Wenzel P, Gori T, Dimitriadis Z (2022) Use of intravascular ultrasound for optimal vessel sizing in chronic total occlusion percutaneous coronary intervention. Front Cardiovasc Med 9:922366 35990972 10.3389/fcvm.2022.922366 PMC9381831

[4] Blessing R, Keller K, Dimitriadis Z, Munzel T, Gori T, Hobohm L (2024) Temporal trends of case-fatality in patients undergoing dual-injection coronary chronic total occlusion recanalization. Clin Res Cardiol 113(7):987–994 37695528 10.1007/s00392-023-02298-x PMC11219465

[5] Di Mario C, Mashayekhi KA, Garbo R, Pyxaras SA, Ciardetti N, Werner GS (2022) Recanalisation of coronary chronic total occlusions. EuroIntervention 18(7):535–561 36134683 10.4244/EIJ-D-21-01117

[6] Sapontis J, Salisbury AC, Yeh RW, Cohen DJ, Hirai T, Lombardi W, McCabe JM, Karmpaliotis D, Moses J, Nicholson WJ, Pershad A, Wyman RM, Spaedy A, Cook S, Doshi P, Federici R, Thompson CR, Marso SP, Nugent K, Gosch K, Spertus JA, Grantham JA, 2017 Early procedural and health status outcomes after chronic total occlusion angioplasty: a report from the OPEN-CTO registry (Outcomes, Patient Health Status, and Efficiency in Chronic Total Occlusion Hybrid Procedures) JACC Cardiovasc Interv 10(15):1523–1534 10.1016/j.jcin.2017.05.065 28797429

[7] Werner GS, Hildick-Smith D, Martin Yuste V, Boudou N, Sianos G, Gelev V, Rumoroso JR, Erglis A, Christiansen EH, Escaned J, Di Mario C, Teruel L, Bufe A, Lauer B, Galassi AR, Louvard Y (2023) Three-year outcomes of a randomized multicentre trial comparing revascularization and optimal medical therapy for chronic total coronary occlusions (EuroCTO). EuroIntervention 19(7):571–9 37482940 10.4244/EIJ-D-23-00312 PMC10493774

[8] Werner GS, Martin-Yuste V, Hildick-Smith D, Boudou N, Sianos G, Gelev V, Rumoroso JR, Erglis A, Christiansen EH, Escaned J, di Mario C, Hovasse T, Teruel L, Bufe A, Lauer B, Bogaerts K, Goicolea J, Spratt JC, Gershlick AH, Galassi AR, Louvard Y (2018) A randomized multicentre trial to compare revascularization with optimal medical therapy for the treatment of chronic total coronary occlusions. Eur Heart J 39(26):2484–93 29722796 10.1093/eurheartj/ehy220

[9] Obedinskiy AA, Kretov EI, Boukhris M, Kurbatov VP, Osiev AG, Ibn Elhadj Z, Obedinskaya NR, Kasbaoui S, Grazhdankin IO, Prokhorikhin AA, Zubarev DD, Biryukov A, Pokushalov E, Galassi AR, Baystrukov VI (2018) The IMPACTOR-CTO Trial. JACC Cardiovasc Interv 11(13):1309–11 29976368 10.1016/j.jcin.2018.04.017

[10] Borgia F, Viceconte N, Ali O, Stuart-Buttle C, Saraswathyamma A, Parisi R, Mirabella F, Dimopoulos K, Di Mario C (2012) Improved cardiac survival, freedom from MACE and angina-related quality of life after successful percutaneous recanalization of coronary artery chronic total occlusions. Int J Cardiol 161(1):31–8 21722979 10.1016/j.ijcard.2011.04.023

[11] Jones DA, Weerackody R, Rathod K, Behar J, Gallagher S, Knight CJ, Kapur A, Jain AK, Rothman MT, Thompson CA, Mathur A, Wragg A, Smith EJ (2012) Successful recanalization of chronic total occlusions is associated with improved long-term survival. JACC Cardiovasc Interv 5(4):380–8 22516393 10.1016/j.jcin.2012.01.012

[12] Lee SW, Lee PH, Ahn JM, Park DW, Yun SC, Han S, Kang H, Kang SJ, Kim YH, Lee CW, Park SW, Hur SH, Rha SW, Her SH, Choi SW, Lee BK, Lee NH, Lee JY, Cheong SS, Kim MH, Ahn YK, Lim SW, Lee SG, Hiremath S, Santoso T, Udayachalerm W, Cheng JJ, Cohen DJ, Muramatsu T, Tsuchikane E, Asakura Y, Park SJ (2019) Randomized trial evaluating percutaneous coronary intervention for the treatment of chronic total occlusion. Circulation 139(14):1674–83 30813758 10.1161/CIRCULATIONAHA.118.031313

[13] Henriques JP, Hoebers LP, Råmunddal T, Laanmets P, Eriksen E, Bax M, Ioanes D, Suttorp MJ, Strauss BH, Barbato E, Nijveldt R, van Rossum AC, Marques KM, Elias J, van Dongen IM, Claessen BE, Tijssen JG, van der Schaaf RJ (2016) Percutaneous intervention for concurrent chronic total occlusions in patients with STEMI: the EXPLORE trial. J Am Coll Cardiol 68(15):1622–32 27712774 10.1016/j.jacc.2016.07.744

[14] Mashayekhi K, Nührenberg TG, Toma A, Gick M, Ferenc M, Hochholzer W, Comberg T, Rothe J, Valina CM, Löffelhardt N, Ayoub M, Zhao M, Bremicker J, Jander N, Minners J, Ruile P, Behnes M, Akin I, Schäufele T, Neumann FJ, Büttner HJ (2018) A Randomized trial to assess regional left ventricular function after stent implantation in chronic total occlusion: the REVASC trial. JACC Cardiovasc Interv 11(19):1982–91 30219327 10.1016/j.jcin.2018.05.041

[15] Scarsini R, Shanmuganathan M, Kotronias RA, Terentes-Printzios D, Borlotti A, Langrish JP, Lucking AJ, OxAMI Study Investigators, Ribichini F, Ferreira VM, Channon KM, Garcia-Garcia HM, Banning AP, De Maria GL (2021) Angiography-derived index of microcirculatory resistance (IMR(angio)) as a novel pressure-wire-free tool to assess coronary microvascular dysfunction in acute coronary syndromes and stable coronary artery disease. Int J Cardiovasc Imaging 37(6):1801–13 33950329 10.1007/s10554-021-02254-8

[16] Konijnenberg LSF, Damman P, Duncker DJ, Kloner RA, Nijveldt R, van Geuns RM, Berry C, Riksen NP, Escaned J, van Royen N (2020) Pathophysiology and diagnosis of coronary microvascular dysfunction in ST-elevation myocardial infarction. Cardiovasc Res 116(4):787–805 31710673 10.1093/cvr/cvz301 PMC7061278

[17] De Maria GL, Scarsini R, Shanmuganathan M, Kotronias RA, Terentes-Printzios D, Borlotti A, Langrish JP, Lucking AJ, Choudhury RP, Kharbanda R, Ferreira VM, Oxford Acute Myocardial Infarction (OXAMI) Study Investigators, Channon KM, Garcia-Garcia HM, Banning AP (2020) Angiography-derived index of microcirculatory resistance as a novel, pressure-wire-free tool to assess coronary microcirculation in ST elevation myocardial infarction. Int J Cardiovasc Imaging 36(8):1395–406 32409977 10.1007/s10554-020-01831-7 PMC7381481

[18] Dai N, Che W, Liu L, Zhang W, Yin G, Xu B, Xu Y, Duan S, Yu H, Li C, Yao K, Huang D, Ge J (2021) Diagnostic value of angiography-derived IMR for coronary microcirculation and its prognostic implication after PCI. Front Cardiovasc Med 8:735743 34722667 10.3389/fcvm.2021.735743 PMC8553988

[19] Choi KH, Dai N, Li Y, Kim J, Shin D, Lee SH, Joh HS, Kim HK, Jeon KH, Ha SJ, Kim SM, Jang MJ, Park TK, Yang JH, Song YB, Hahn JY, Doh JH, Shin ES, Choi SH, Gwon HC, Lee JM (2021) Functional coronary angiography-derived index of microcirculatory resistance in patients with st-segment elevation myocardial infarction. JACC Cardiovasc Interv 14(15):1670–84 34353599 10.1016/j.jcin.2021.05.027

[20] Castaldi G, Fezzi S, Widmann M, Lia M, Rizzetto F, Mammone C, Pazzi S, Piccolo S, Galli V, Pighi M, Pesarini G, Prati D, Ferrero V, Scarsini R, Tavella D, Ribichini F (2023) Angiography-derived index of microvascular resistance in takotsubo syndrome. Int J Cardiovasc Imaging 39(1):233–44 36336756 10.1007/s10554-022-02698-6 PMC9813145

[21] Mejia-Renteria H, Lee JM, Choi KH, Lee SH, Wang L, Kakuta T, Koo BK, Escaned J (2021) Coronary microcirculation assessment using functional angiography: development of a wire-free method applicable to conventional coronary angiograms. Catheter Cardiovasc Interv 98(6):1027–37 34242489 10.1002/ccd.29863

[22] Zhou J, Onuma Y, Garg S, Kotoku N, Kageyama S, Masuda S, Ninomiya K, Huo Y, Reiber JHC, Tu S, Piek JJ, Escaned J, Perera D, Bourantas C, Yan H, Serruys W, P, (2022) Angiography derived assessment of the coronary microcirculation: is it ready for prime time? Expert Rev Cardiovasc Ther 20(7):549–66 35899781 10.1080/14779072.2022.2098117

[23] Ladwiniec A, Cunnington MS, Rossington J, Thackray S, Alamgir F, Hoye A (2016) Microvascular dysfunction in the immediate aftermath of chronic total coronary occlusion recanalization. Catheter Cardiovasc Interv 87(6):1071–9 26756537 10.1002/ccd.26392

[24] Werner GS, Surber R, Ferrari M, Fritzenwanger M, Figulla HR (2006) The functional reserve of collaterals supplying long-term chronic total coronary occlusions in patients without prior myocardial infarction. Eur Heart J 27(20):2406–12 17003048 10.1093/eurheartj/ehl270

[25] Werner GS, Surber R, Kuethe F, Emig U, Schwarz G, Bahrmann P, Figulla HR (2005) Collaterals and the recovery of left ventricular function after recanalization of a chronic total coronary occlusion. Am Heart J 149(1):129–37 15660044 10.1016/j.ahj.2004.04.042

[26] Werner GS, Fritzenwanger M, Prochnau D, Schwarz G, Ferrari M, Aarnoudse W, Pijls NH, Figulla HR (2006) Determinants of coronary steal in chronic total coronary occlusions donor artery, collateral, and microvascular resistance. J Am Coll Cardiol 48(1):51–8 16814648 10.1016/j.jacc.2005.11.093

[27] Li M, Su H, Zuo Z, Zhang Z, Li M, Su F, Yao W, He Y, Kong X, Wang H (2023) The role of the angiography-derived index of microcirculatory resistance in the prognosis of patients with dilated cardiomyopathy. Quant Imaging Med Surg. 13(4):2647–59 37064388 10.21037/qims-22-1060 PMC10102743

[28] Mohammed AQ, Abdu FA, Su Y, Liu L, Yin G, Feng Y, Zhang W, Xu Y, Xu D, Che W (2023) Prognostic significance of coronary microvascular dysfunction in patients with heart failure with preserved ejection fraction. Can J Cardiol 39(7):971–80 37086837 10.1016/j.cjca.2023.04.011

[29] Brilakis ES, Grantham JA, Rinfret S, Wyman RM, Burke MN, Karmpaliotis D, Lembo N, Pershad A, Kandzari DE, Buller CE, DeMartini T, Lombardi WL, Thompson CA (2012) A percutaneous treatment algorithm for crossing coronary chronic total occlusions. JACC Cardiovasc Interv 5(4):367–79 22516392 10.1016/j.jcin.2012.02.006

[30] Ullrich-Daub H, Daub S, Olschewski M, Munzel T, Gori T (2023) Diseases of the coronary microcirculation: diagnosis and treatment. Dtsch Arztebl Int 120(44):739–46 37721132 10.3238/arztebl.m2023.0205 PMC10722490

[31] Fearon WF, Balsam LB, Farouque HM, Caffarelli AD, Robbins RC, Fitzgerald PJ, Yock PG, Yeung AC (2003) Novel index for invasively assessing the coronary microcirculation. Circulation 107(25):3129–32 12821539 10.1161/01.CIR.0000080700.98607.D1

[32] Ng MK, Yeung AC, Fearon WF (2006) Invasive assessment of the coronary microcirculation: superior reproducibility and less hemodynamic dependence of index of microcirculatory resistance compared with coronary flow reserve. Circulation 113(17):2054–61 16636168 10.1161/CIRCULATIONAHA.105.603522

[33] Hong D, Shin D, Lee SH, Joh HS, Choi KH, Kim HK, Ha SJ, Park TK, Yang JH, Song YB, Hahn JY, Choi SH, Gwon HC, Lee JM (2023) Prognostic impact of coronary microvascular dysfunction according to different patterns by invasive physiologic indexes in symptomatic patients with intermediate coronary stenosis. Circ Cardiovasc Interv 16(3):e012621 36846961 10.1161/CIRCINTERVENTIONS.122.012621

[34] Keulards DCJ, Karamasis GV, Alsanjari O, Demandt JPA, Van’t Veer M, Zelis JM, Dello SA, El Farissi M, Konstantinou K, Tang KH, Kelly PA, Keeble TR, Pijls NHJ, Davies JR, Teeuwen K (2020) Recovery of absolute coronary blood flow and microvascular resistance after chronic total occlusion percutaneous coronary intervention: an exploratory study. J Am Heart Assoc 9(9):e015669 32316813 10.1161/JAHA.119.015669 PMC7428549

[35] Khan SA, Alsanjari O, Keulards DCJ, Vlaar PJ, Zhang J, Konstantinou K, Fawaz S, Simpson R, Clesham G, Kelly PA, Tang KH, Cook CM, Cockburn J, Pijls NHJ, Hildick-Smith D, Teeuwen K, Keeble TR, Karamasis GV, Davies JR (2023) Changes in absolute flow, myocardial resistance and FFR after chronic total occlusion percutaneous coronary intervention. EuroIntervention 19(2):e123–e33 36722201 10.4244/EIJ-D-22-00694 PMC10242660

[36] Zhang QX, Gao SY, Fang S, Fan FF, Yang F, Zhou ZY, Zheng B, Gong YJ (2025) The prognostic value of coronary angiography-derived index of microcirculatory resistance in patients who underwent the percutaneous coronary intervention. Zhonghua Xin Xue Guan Bing Za Zhi 53(5):505–13 40389340 10.3760/cma.j.cn112148-20250123-00065

[37] Westra J, Andersen BK, Campo G, Matsuo H, Koltowski L, Eftekhari A, Liu T, Di Serafino L, Di Girolamo D, Escaned J, Nef H, Naber C, Barbierato M, Tu S, Neghabat O, Madsen M, Tebaldi M, Tanigaki T, Kochman J, Somi S, Esposito G, Mercone G, Mejia-Renteria H, Ronco F, Bøtker HE, Wijns W, Christiansen EH, Holm NR (2018) diagnostic performance of in-procedure angiography-derived quantitative flow reserve compared to pressure-derived fractional flow reserve: the FAVOR II Europe-Japan study. J Am Heart Assoc 7(14):009603 10.1161/JAHA.118.009603 PMC6064860 29980523

[38] Zhang Y, Pu J, Niu T, Fang J, Chen D, Yidilisi A, Zheng Y, Lu J, Hu Y, Koo BK, Xiang J, Wang J, Jiang J (2024) Prognostic value of coronary angiography-derived index of microcirculatory resistance in non-st-segment elevation myocardial infarction patients. JACC Cardiovasc Interv 17(16):1874–86 39115479 10.1016/j.jcin.2024.04.048

[39] El Farissi M, Zimmermann FM, De Maria GL, van Royen N, van Leeuwen MAH, Carrick D, Carberry J, Wijnbergen IF, Konijnenberg LSF, Hoole SP, Marin F, Fineschi M, Pijls NHJ, Oldroyd KG, Banning AP, Berry C, Fearon WF (2023) The index of microcirculatory resistance after primary pci: a pooled analysis of individual patient data. JACC Cardiovasc Interv 16(19):2383–92 37821183 10.1016/j.jcin.2023.08.030

[40] Schweiger V, Gilhofer T, Fang R, Candreva A, Seifert B, Di Vece D, Wuerdinger M, Koleva I, Rajman K, Cieslik M, Gotschy A, Michel J, Stehli J, Niederseer D, Ryberg L, Ghadri J, Ruschitzka F, Stähli B, Cammann VL, Templin C (2024) Coronary microvascular dysfunction in Takotsubo syndrome: an analysis using angiography-derived index of microcirculatory resistance. Clin Res Cardiol. 113(12):1629–37 37985475 10.1007/s00392-023-02329-7 PMC11579140

[41] Nishi T, Murai T, Ciccarelli G, Shah SV, Kobayashi Y, Derimay F, Waseda K, Moonen A, Hoshino M, Hirohata A, Yong ASC, Ng MKC, Amano T, Barbato E, Kakuta T, Fearon WF (2019) Prognostic value of coronary microvascular function measured immediately after percutaneous coronary intervention in stable coronary artery disease: an international multicenter study. Circ Cardiovasc Interv 12(9):e007889 31525096 10.1161/CIRCINTERVENTIONS.119.007889

[42] Sans-Roselló J, Fernández-Peregrina E, Duran-Cambra A, Carreras-Mora J, Sionis A, Álvarez-García J, García-García HM (2022) Prognostic value of microvascular resistance at rest in patients with Takotsubo syndrome. JACC Cardiovasc Imaging 15(10):1784–95 36125887 10.1016/j.jcmg.2022.03.030

[43] Del Buono MG, Montone RA, Camilli M, Carbone S, Narula J, Lavie CJ, Niccoli G, Crea F (2021) Coronary microvascular dysfunction across the spectrum of cardiovascular diseases: JACC State-of-the-Art review. J Am Coll Cardiol 78(13):1352–71 34556322 10.1016/j.jacc.2021.07.042 PMC8528638

[44] Taqueti VR, Di Carli MF (2018) Coronary microvascular disease pathogenic mechanisms and therapeutic options: JACC State-of-the-Art review. J Am Coll Cardiol 72(21):2625–41 30466521 10.1016/j.jacc.2018.09.042 PMC6296779

[45] Smilowitz NR, Toleva O, Chieffo A, Perera D, Berry C (2023) Coronary Microvascular disease in contemporary clinical practice. Circ Cardiovasc Interv 16(6):e012568 37259860 10.1161/CIRCINTERVENTIONS.122.012568 PMC10330260

[46] Padro T, Manfrini O, Bugiardini R, Canty J, Cenko E, De Luca G, Duncker DJ, Eringa EC, Koller A, Tousoulis D, Trifunovic D, Vavlukis M, de Wit C, Badimon L (2020) ESC Working Group on Coronary Pathophysiology and Microcirculation position paper on “coronary microvascular dysfunction in cardiovascular disease.” Cardiovasc Res 116(4):741–55 32034397 10.1093/cvr/cvaa003 PMC7825482

[47] Fan Y, Fezzi S, Sun P, Ding N, Li X, Hu X, Wang S, Wijns W, Lu Z, Tu S (2022) In vivo validation of a novel computational approach to assess microcirculatory resistance based on a single angiographic view. J Pers Med 12(11):1798 36573725 10.3390/jpm12111798 PMC9692562

[48] Mejía-Rentería H, Wang L, Chipayo-Gonzales D, van de Hoef TP, Travieso A, Espejo C, Núñez-Gil IJ, Macaya F, Gonzalo N, Escaned J (2023) Angiography-derived assessment of coronary microcirculatory resistance in patients with suspected myocardial ischaemia and non-obstructive coronary arteries. EuroIntervention 18(16):e1348–e56 36534493 10.4244/EIJ-D-22-00579 PMC10068857

[49] Keulards DCJ, Vlaar PJ, Wijnbergen I, Pijls NHJ, Teeuwen K (2021) Coronary physiology before and after chronic total occlusion treatment: what does it tell us? Neth Heart J 29(1):22–9 32720123 10.1007/s12471-020-01470-6 PMC7782651

[50] Sagris M, Theofilis P, Antonopoulos AS, Oikonomou E, Paschaliori C, Galiatsatos N, Tsioufis K, Tousoulis D (2021) Inflammation in coronary microvascular dysfunction. Int J Mol Sci 22(24):13471 34948272 10.3390/ijms222413471 PMC8703507

[51] Schindler TH, Dilsizian V (2020) Coronary microvascular dysfunction: clinical considerations and noninvasive diagnosis. JACC Cardiovasc Imaging 13(1 Pt 1):140–55 30982670 10.1016/j.jcmg.2018.11.036

[52] Shaw LJ, Berman DS, Maron DJ, Mancini GB, Hayes SW, Hartigan PM, Weintraub WS, O’Rourke RA, Dada M, Spertus JA, Chaitman BR, Friedman J, Slomka P, Heller GV, Germano G, Gosselin G, Berger P, Kostuk WJ, Schwartz RG, Knudtson M, Veledar E, Bates ER, McCallister B, Teo KK, Boden WE (2008) Optimal medical therapy with or without percutaneous coronary intervention to reduce ischemic burden: results from the clinical outcomes utilizing revascularization and aggressive drug evaluation (COURAGE) trial nuclear substudy. Circulation 117(10):1283–91 18268144 10.1161/CIRCULATIONAHA.107.743963

[53] Yu H, Kalogeris T, Korthuis RJ (2019) Reactive species-induced microvascular dysfunction in ischemia/reperfusion. Free Radic Biol Med 135:182–97 30849489 10.1016/j.freeradbiomed.2019.02.031 PMC6503659

[54] Allahwala UK, Weaver J, Bhindi R (2019) Animal chronic total occlusion models: a review of the current literature and future goals. Thromb Res 177:83–90 30856383 10.1016/j.thromres.2019.03.004

[55] Konishi T, Kawakami R, Vozenilek AE, Ghosh SKB, Xu W, Grogan A, Shah P, Tanaka T, Sekimoto T, Shiraki T, Kawai K, Sato Y, Mori M, Sakamoto A, Hisadome H, Ashida K, Bellissard A, Williams D, Dryanovski D, Kutys R, Cheng Q, Romero M, Chahal D, Virmani R, Finn AV (2024) Mechanisms of medial wall thinning in chronic total occlusion. JACC Cardiovasc Interv 17(14):1719–28 38970581 10.1016/j.jcin.2024.05.013

[56] Geyer M, Wild J, Hirschmann M, Dimitriadis Z, Münzel T, Gori T, Wenzel P (2020) Predictors for target vessel failure after recanalization of chronic total occlusions in patients undergoing surveillance coronary angiography. J Clin Med 9(1):178 31936478 10.3390/jcm9010178 PMC7019748

[57] Harkin KL, Loftspring E, Beaty W, Joa A, Serrano-Gomez C, Farid A, Hausvater A, Reynolds HR, Smilowitz NR (2024) Visual estimates of coronary slow flow are not associated with invasive wire-based diagnoses of coronary microvascular dysfunction. Circ Cardiovasc Interv 17(6):e013902 38583174 10.1161/CIRCINTERVENTIONS.123.013902 PMC11187652

